# FTY720 Protects Against Ischemia–Reperfusion Injury by Preventing the Redistribution of Tight Junction Proteins and Decreases Inflammation in the Subacute Phase in an Experimental Stroke Model

**DOI:** 10.1007/s12975-020-00789-x

**Published:** 2020-02-27

**Authors:** Zifeng Wang, Kei Higashikawa, Hironobu Yasui, Yuji Kuge, Yusuke Ohno, Akio Kihara, Yenari A. Midori, Kiyohiro Houkin, Masahito Kawabori

**Affiliations:** 1 Department of Neurosurgery, Graduate School of Medicine, Hokkaido University, Kita 15, Nishi 7, Kita-ku, Sapporo, Hokkaido 060-8638 Japan; 2 Central Institutes of Isotope Science (Laboratory of Integrated Molecular Imaging, Department of Biomedical Imaging, Graduate School of Biomedical Science and Engineering), Hokkaido University, Sapporo, Hokkaido Japan; 3 Laboratory of Biochemistry, Faculty of Pharmaceutical Sciences, Hokkaido University, Sapporo, Hokkaido Japan; 4 Department of Neurology, University of California, San Francisco and the San Francisco Veterans Affairs Medical Center, San Francisco, CA USA

**Keywords:** FTY720, Sphingosine-1-phosphate receptor, Brain ischemia–reperfusion, Blood–brain barrier, Inflammation, Apoptosis

## Abstract

Injury due to brain ischemia followed by reperfusion (I/R) may be an important therapeutic target in the era of thrombectomy. FTY720, a widely known sphingosine-1-phosphate receptor agonist, exerts various neuroprotective effects. The aim of this study was to examine the protective effect of FTY720 with respect to I/R injury, especially focusing on blood–brain barrier (BBB) protection and anti-inflammatory effects. Male rats were subjected to transient ischemia and administered vehicle or 0.5 or 1.5 mg/kg of FTY720 immediately before reperfusion. Positron emission tomography (PET) with [^18^F]DPA-714 was performed 2 and 9 days after the insult to serially monitor neuroinflammation. Bovine and rat brain microvascular endothelial cells (MVECs) were also subjected to oxygen-glucose deprivation (OGD) and reperfusion, and administered FTY720, phosphorylated-FTY720 (FTY720-P), or their inhibitor. FTY720 dose-dependently reduced cell death, the infarct size, cell death including apoptosis, and inflammation. It also ameliorated BBB disruption and neurological deficits compared to in the vehicle group. PET indicated that FTY720 significantly inhibited the worsening of inflammation in later stages. FTY720-P significantly prevented the intracellular redistribution of tight junction proteins but did not increase their mRNA expression. These results suggest that FTY720 can ameliorate I/R injury by protecting the BBB and regulating neuroinflammation.

**Electronic supplementary material:**

The online version of this article (10.1007/s12975-020-00789-x) contains supplementary material, which is available to authorized users.

## Introduction

Ischemic stroke is a major health problem worldwide and is associated with extremely high morbidity and mortality [1]. Recent advances in thrombectomy have brought forth a new era for the treatment of acute ischemic stroke [2–4]. Thrombectomy can achieve higher rates of angiographic revascularization and provides better functional outcomes even up to 24 h after symptom onset. Although recanalization by thrombectomy can restore cerebral blood flow, many patients still do not show sufficient clinical recovery [2–4]. Ischemia–reperfusion (I/R) injury is thought to be one of the major reasons for unfavorable outcomes following thrombolysis and thrombectomy. I/R injury is defined as a biochemical cascade that exacerbates damage in the re-perfused brain tissue [5]. It can affect pathways leading to excitotoxicity, apoptosis, inflammation, free radical production, and loss of blood–brain barrier (BBB) integrity. These insults subsequently induce immune cell infiltration and extracellular matrix damage, resulting in brain edema and hemorrhagic transformation [6]. Thus, studies are urgently needed to understand these mechanisms and treatments to maximize the therapeutic effect of thrombectomy. FTY720, a novel immunomodulator that acts as a sphingosine-1-phosphate (S1P) receptor agonist, was recently approved for the treatment for multiple sclerosis by the US Food and Drug Administration. In vivo, FTY720 can be rapidly phosphorylated, generating its biologically active form, namely phosphorylated-FTY720 (FTY720-P), which subsequently binds four of the five S1P receptors to regulate multiple cellular events [7]. The effect of FTY720 includes the induction of systemic lymphopenia, inhibition of inflammatory responses, and downregulation of intravascular adhesion molecules. Recently, several studies have shown that FTY720 protects organs from I/R injury, such as the liver [8, 9], kidney [10, 11], heart [12–14], and brain [15–19]. However, the mechanisms underlying the BBB-protective effects of FTY720 and time course of inflammatory modulation have not been fully elucidated. The aim of this study was to examine the protective effect of FTY720 on brain I/R, with a focus on BBB preservation and the regulation of inflammatory reactions.

## Materials and Methods

All experimental protocols were approved by the Animal Studies Ethical Committee at Hokkaido University Graduate School of Medicine (reference number 17-0066). All procedures used in the present study were performed in accordance with the institutional guidelines for animal experimentation and the Guidelines for Proper Conduct of Animal Experiments by the Science Council of Japan and the ARRIVE (Animal Research: Reporting In Vivo Experiments) guidelines.

### Experimental Animals

In total, 123 wild-type 8-week-old male Sprague-Dawley rats (CLEA Japan, Inc., Tokyo, Japan) weighing 260–300 g were used for the experiments. Male rats were employed to eliminate any estrogen-mediated neuroprotective effects on ischemic injury. Animals were housed in a controlled environment (25 °C, 50% humidity, and a 12-h light–dark cycle) and allowed free access to food and water. All experimental animals were randomly divided into the following groups: middle cerebral artery occlusion (MCAO) with vehicle treatment (*n* = 60), MCAO with low-dose (0.5 mg/kg) FTY720 treatment (*n* = 29), and MCAO with high-dose of FTY720 (1.5 mg/kg) treatment (*n* = 28).

### tMCAO Model

Transient focal cerebral ischemia was induced for 2 h using a silicone rubber-coated nylon filament inserted into the right middle cerebral artery (MCA) as previously described [20–22]. Briefly, rats were anesthetized with isoflurane at initial and maintenance concentrations of 4.0% and 2.0%, respectively, in 70% N_2_O and 30% O_2_ gas, thorough a facial mask. The rectal temperature of experimental animals was maintained between 36.5 and 37.5 °C throughout the procedures using an automated heat pad. Transient focal ischemia was induced by occluding the right MCA using a silicone rubber-coated nylon filament with a tip diameter of 0.37 mm (Doccol Corp., Redlands, CA, USA). The right carotid artery (CA) was surgically exposed, and the external CA was then ligated, cut, and reversed proximally. The filament was inserted into the external CA and advanced into the internal CA to block the origin of the MCA. Cerebral blood flow (CBF) measurements and neurological scoring assessments were performed to verify successful MCAO. CBF in the MCA region (5 mm lateral to the middle line and 2 mm posterior to the bregma) was measured before and after MCAO by laser Doppler flowmetry (OMEGA FLOW FLO-C1; OMEGAWAVE, Tokyo, Japan). Rats with a relative reduction of CBF higher than 30% compared to preoperative data and/or a modified Bederson score of 2 points or lower were excluded from the experiment. After the 2-h transient middle cerebral artery occlusion (tMCAO), the filament was gently withdrawn to provide reperfusion.

### Administration of FTY720

The low-dose group was administered 0.5 mg/kg FTY720 (Cayman Chemical, Ann Arbor, MI, USA) diluted in saline, whereas the high-dose group was treated with 1.5 mg/kg FTY720. FTY720 was intraperitoneally injected immediately before reperfusion.

### Neurological Score

An 18-point modified neurological severity score (mNSS) and four-point modified Bederson score were used to evaluate neurological deficits [23, 24]. Neurological evaluation was performed before and after MCA occlusion on days 1, 3, 5, and 7.

### Evaluation of Brain Infarct Volume

The infarct volume was evaluated 7 days after reperfusion by 2,3,5-triphenyltetrazolium chloride (TTC) staining. Rats were deeply anesthetized to prevent pain and discomfort at the time of sacrifice. They were then sacrificed by spinal dislocation and decapitation 7 days after reperfusion. The brains were then removed and placed immediately on ice for 5 min. Brain samples were sliced into six uniform coronal sections of 2-mm thickness, and sections were stained with 2% TTC for 15 min [25, 26]. The infarct lesions were photographed and quantitatively analyzed with ImageJ software (ImageJ 1.37v; NIH, Bethesda, MD, USA) by an observer blinded to the groups. Unstained areas (pale color) were defined as ischemic lesions, and the infarct size was calculated based on the following formula to avoid the effect of brain edema [27].

Brain infarct (%) = (contralateral hemispheric volume − ipsilateral non-infarct volume)/contralateral hemispheric volume.

### Immunohistochemistry and Apoptosis Assay

Paraffin sections of brain tissue fixed in 4% paraformaldehyde were used for immunohistochemistry. Four-micrometer-thick coronal sections at the level of the striatum were prepared. The antibodies used for immunohistochemistry of neuroinflammation were anti-Iba1 (1:1500 at room temperature for 1 h; 019-19741, Wako, Japan) and anti-CD68 (1:1000 at room temperature for 1 h; MCA341GA, BIO-RAD). After incubation with the primary antibody, the sections were treated with Histofine® Simple StainTM Rat MAX PO (Nichirei Biosciences, Tokyo, Japan) for 1 h. Next, the DAB chromogen of the DAB Substitute Kit (Nichirei Biosciences) was applied for 10–30 s to obtain the chromogenic signal in accordance with the manufacturer’s instructions. For immunofluorescence staining of apoptotic cells, TUNEL was performed using the ApopTag Fluorescein In Situ Apoptosis Detection Kit (S7110, Chemicon International, Temecula, CA, USA) according to the manufacturer’s protocol. Ten regions of interest (ROIs) (100 × 100 μm) were randomly placed on the area adjacent to the boundary zone of the infarct (peri-infarct) to count the positive cells [28].

### PET Scans and Autoradiography Imaging

A neural inflammatory biomarker translocator protein (TSPO) was serially monitored on days 2 and 9 by positron emission tomography (PET) using the TSPO ligand [^18^F]DPA-714 as previously reported [29]. PET imaging was serially performed with the same rats (*n* = 4 per group); images were obtained using the Inveon small animal imaging system (Siemens Medical Solutions, Knoxville, TN, USA). The PET component consisted of 1.5 × 1.5 × 10 mm lutetium oxyorthosilicate crystal elements with a ring diameter of 16.1 cm, providing a 10-cm transaxial and 12.7-cm axial field of view [30]. Rats anesthetized with isoflurane were injected with 13.2 ± 0.8 MBq [18F]DPA-714 via the tail vein. They were then returned to their cages and allowed to move freely for 30 min. All performance measurements were set to a coincidence window of 3.432 ns and energy window of 350–650 keV. Delayed events were subtracted from prompt events to correct for random events. The image matrix was 256 × 256 × 159, resulting in a voxel size of 0.385 × 0.385 × 0.796 mm (512 × 512 × 159 matrix, 0.215 × 0.215 × 0.796 mm for spatial resolution measurements) [31]. ROI analysis was conducted in the area of infarction, and the control was symmetrically placed in the contralateral hemisphere. Next, counting rates were converted to standardized uptake values (SUVs) and the ratio of ipsilateral to contralateral radioactivity was calculated using IDL (Research Systems, CO, USA) and ASIPro VM (Concorde Microsystems, Knoxville, TN, USA). For autoradiography experiments, rats were sacrificed at 90 min after [18F]DPA-714 injection. The brains were quickly removed and cut into six 2-mm-thick coronal slices. The second and fourth slices were exposed to a phosphor imaging plate (Fuji Photo Film Co., Ltd., Tokyo, Japan) together with a set of calibrated standards. After exposure, the imaging plate was scanned with an FLA 7000 BioImaging Analyzer (Fujifilm Life Science) and images were analyzed using Multi Gauge V3.2 (Fujifilm Life Science).

### Evaluation of In Vivo BBB Integrity

Evans blue dye and fluorescent dextran were used to measure BBB disruption [21, 32]. When intravenously injected, Evans blue binds to albumin to form a high molecular weight serum protein complex (68 kDa), and two different types of fluorescent dextran (3 and 10 kDa) were also applied to determine the size selectivity of BBB leakage. Immediately after reperfusion, 4 mL/kg of 2% Evans blue (Wako, Osaka, Japan) or 50 mg/kg fluorescent dextran (FD4; 3 kD, FD10S; 10 kD, Sigma, St. Louis, MO, USA) diluted in saline was intravenously administered. The amount of Evans blue leakage was assessed by brain supernatant absorbance, and fluorescent dextran was assessed by immunohistochemistry analysis as previously reported [21, 32]. Briefly, at 24 h after reperfusion, the rats were sacrificed and perfused with saline through the left ventricle to wash out the intravascular Evans blue or fluorescent dextran. The right cerebral hemisphere was then removed and homogenized in 3 mL of 50% trichloroacetic acid solution and then centrifuged (12,000 rpm for 10 min). The absorbance of the supernatant at 620 nm was measured using a spectrophotometer (TECAN Japan, Kanagawa, Japan). The tissue contents of Evans blue were quantified from a linear standard curve and expressed as micrograms per gram of brain tissue. Fluorescent dextran leakage was measured by immunohistochemistry. Four-micrometer-thick paraffin coronal sections at the level of striatum were prepared. The sections were treated with a collagen IV antibody (1:200, 4 °C overnight, Proteintech, Rosemont, IL, USA) followed by Alexa Fluor 594 Goat Anti-Rabbit IgG Antibody (1:200, A11012, Invitrogen). Extra-vascular dextran leakage was assessed in randomly selected 5 non-overlapping fields (700 × 560 μm) at around the peri-infarct area. The amount of leakage was semi-quantitatively analyzed by the following formula: vascular leakage = area of leakage (fluorescent dextran)/microvascular area (collagen IV).

### Cell Culture

Bovine brain microvascular endothelial cells (BBMVECs) were purchased from Cell Applications, Inc. (San Diego, CA, USA), and the rat microvascular BBB kit was purchased from PharmaCo-Cell, Inc. (Nagasaki, Japan) for in vitro experiments. Next, 100-mm cell culture dishes (Thermo Fisher Scientific, Waltham, MA, USA), 2-well chamber slides (Watson Biolab, Kobe, Japan), and a permeability kit (PharmaCo-Cell) were used to culture cells in high-glucose Dulbecco’s modified Eagle’s medium (DMEM; Nacalai Tesque, Inc., Kyoto, Japan), containing 10% fetal bovine serum (FBS) and 100 U/mL penicillin G. The cells were maintained in an incubator at 37 °C with 5% CO_2_, and subconfluent monolayers of early passage cells (P2–4), or cells condition instructed by manufacture were used for each experiment [29].

### OGD and Reperfusion Model

The cells were exposed to oxygen-glucose deprivation (OGD) for 4 h and then returned to normoxic conditions (37 °C, 5% CO_2_) in high-glucose medium without FBS for 4 h, as previously described [20]. Before OGD, the cells were cultured in high-glucose DMEM with activated charcoal-filtered FBS for 12 h, followed by high-glucose DMEM without FBS for another 12 h to eliminate S1P contained in the FBS. OGD was achieved under hypoxic conditions (1% O_2_, 5% CO_2_, and 94% N_2_ at 37 °C) generated by a hypoxia workstation (InvivoO2 300, Baker Ruskinn, Sanford, ME, USA) and DMEM without glucose. This medium was prepared to be hypoxic by overnight incubation under hypoxic conditions. The cells were then randomly divided into the following groups (*n* = 5 per group): (1) control group, no OGD; (2) vehicle group, OGD without treatment; (3) FTY720 group, OGD with 100 nM of FTY720 added to the culture medium immediately before reperfusion; (4) FTY720-P group, OGD with 100 nM of FTY720-P (Cayman Chemical) added to the culture medium immediately before reperfusion; (5) FTY720-P and pertussis toxin (PTX) group, OGD with FTY720-P (100 nM) and PTX (100 ng/mL, Fujifilm) added to the culture medium immediately before reperfusion. As FTY720 must be converted to FTY720-P to be physiologically active and BBMVECs are thought to not possess sufficient enzymes to convert FTY720 to FTY720-P, FTY720-P was also applied to the cells. PTX is an inhibitor of the Gi-coupled S1P receptor and acts as an antagonist of FTY720 and FTY720-P.

### Evaluation of In Vitro BBB Integrity

A rat microvascular BBB kit (PharmacoCell, Nagasaki, Japan) was used to measure in vitro BBB integrity according to the manufacturer’s instructions. Briefly, the cells were cultured as previously mentioned and transendothelial electrical resistance (TEER) was measured just before starting OGD to ensure that the resistance was higher than 150 Ω, which indicates a sufficient barrier function (data not shown). OGD and reperfusion were performed as previously mentioned, and Evans blue preconjugated with serum albumin or FITC dextran (3 and 5 kD) were added to the upper wells (0.2 mg/200 μL). The medium in the lower well was collected 30 min later. The concentration of Evans blue in the lower well was measured with a spectrophotometer (TECAN, Männedorf, Switzerland) as previously described, and the FITC concentration was measured with a fluorescence plate reader (Spectra Max paradigm, Molecular Devices, Sunnyvale, CA, USA). The permeant diffusivity of Evans blue or FITC dextran was measured, and the relative ratio of diffusion was qualitatively analyzed between groups as previously reported [33].

### Real-Time PCR and Western Blotting Analysis

The expression of tight and adheres junctional proteins including ZO-1, occludin, claudin-5, and VE-cadherin after OGD treatment was examined by real-time PCR. RNA was extracted from the cells using an AllPrep RNA Mini Kit (Qiagen, Hilden, Germany). First-strand cDNA was synthesized using a PrimeScript® II 1st strand cDNA Synthesis Kit (Takara, Shiga, Japan) in accordance with the manufacturers’ recommendations. The relative differences in gene expression between groups were expressed based on cycle time (Ct) values. The Ct values of the genes of interest were first normalized to *GAPDH* in the same sample and then relative differences between control and treatment groups were obtained. The primer sequences for real-time PCR are shown in Supplementary Table 1.

Western blotting was performed using an anti-ERK1/2 monoclonal antibody (1:2000, Thermo Fisher Scientific) to evaluate downstream signaling from the S1P_1_ receptor. Proteins were extracted from the BBMECs in 100-mm culture dishes (*n* = 6 per group; control, vehicle, FTY720, FTY720-P, FTY720-P, and PTX). The cells were collected and homogenized in RIPA lysis buffer (Santa Cruz Biotechnology, Dallas, TX, USA) and an equal amount of total protein (10 μg) was electrophoresed on a NuPage 4–12% Bis-Tris Gel (Life Technologies, Carlsbad, CA, USA). Proteins were then blotted onto a cellulose membrane using the iBlot2 (Thermo Fisher Scientific) following the manufacturer’s protocol. The membrane was blocked with ECL Prime blocking agent (GE Healthcare Life Science, Little Chalfont, UK) in PBS containing 0.05% Tween-20 at room temperature for 1 h, followed by incubation with primary antibody at 4 °C overnight. After washing with PBS containing 0.05% Tween-20, the membrane was incubated with a peroxidase-conjugated secondary antibody at room temperature for 1 h. Labeled proteins were detected by chemical luminescence (ECL Advanced Western Blotting Detection Kit; GE Healthcare Life Science). Immunoblots were quantified by densitometry using software provided by the imaging system (ChemiDoc; Bio-Rad, Hercules, CA, USA).

### Immunocytofluorescence of BBB Components

Redistribution of the junctional proteins (ZO-1, VE-cadherin, occludin, claudin-5) was examined by immunocytofluorescence. BBMEVCs or rat brain microvascular endothelial cells were treated with OGD followed by drug administration as previously mentioned. The cells were then fixed with 4% paraformaldehyde for 15 min, permeabilized with 0.1% Triton X-100 for 10 min, and blocked with 3% bovine serum albumin for 30 min. The cells were then incubated with Blockace (DS Pharma Biomedical, Inc., Osaka, Japan) for 1 h to block nonspecific reactions. The cells were treated with anti-VE-cadherin (1:100, catalog number: PA5-17401, Thermo Fisher Scientific), anti-ZO-1 (1:100, catalog number: 33-9100, Thermo Fisher Scientific), anti-occludin (1:100, MBS9608114, MyBioSource), and anti-claudin-5 (1:100, GTX49371, GenTex) as primary antibodies at room temperature for 1 h, followed by incubation with Alexa Fluor 488 Goat Anti-Rabbit IgG Antibody (1:200, Life Technologies) as the secondary antibody at room temperature for 1 h and DAPI staining. Stained images were visualized and photographed with a fluorescence microscope (BZ X-700, Keyence, Osaka, Japan). Ten nonoverlapping fields (700 × 560 μm) were randomly selected, and numbers and ratio of the cells with junctional protein at the cell border or in the cytoplasm were semi-quantitively analyzed [34–36].

### Statistical Analysis

All data were collected and analyzed by investigators blinded to all randomized animal and cell experimental groups. The data are presented as the mean ± standard deviation. All statistical analyses were performed using JMP Pro 13 (Systat Software, San Jose, CA, USA). Continuous and noncontinuous data were compared using an unpaired *t* test or Wilcoxon test between 2 groups and one-way analysis of variance followed by the Dunnett post hoc or Dunn test for multiple comparisons. Sample sizes were selected based on preliminary experiments. Briefly, in one-way analysis of variance study, sample sizes of 10, 10, and 20 were obtained from the 3 groups to compare the means. The total sample of 40 subjects achieves 100% power for detecting differences among the means versus the alternative of equal means using an *F* test with a 0.0500 significance level. The size of the variation in the means is represented by their standard deviation, which was 0.83. The common standard deviation within a group was assumed to be 1.00. We used PASS 14.0.9 (PASS Software by NCSS, LLC) to compute the statistical power. A *p* value < 0.05 was considered as statistically significant.

## Results

### FTY720 Reduces Mortality and Infarct Size and Improves Functional Recovery

**Fig. 1:**
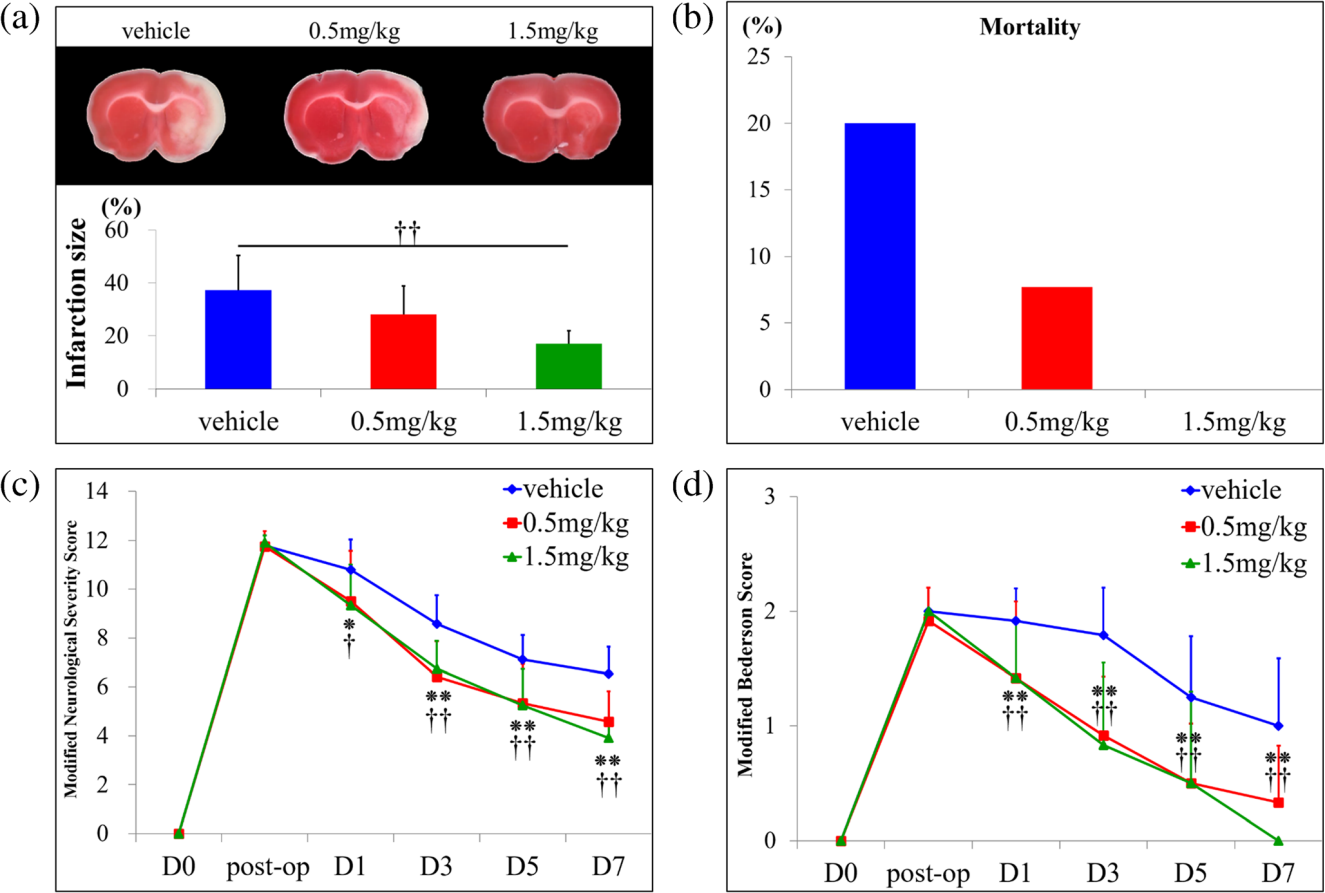
Effect of FTY720 on infarct size, mortality ratio, and neurological recovery in a rat model of transient middle cerebral artery occlusion. **a** Animals (vehicle, *n* = 20; 0.5 mg/kg, *n* = 6; 1.5 mg/kg, n = 6) were sacrificed at day 7 after transient middle cerebral artery occlusion and 2,3,5-triphenyltetrazolium chloride staining of coronal brain sections was performed to analyze the efficacy of FTY720 for reducing the infarct size. High-dose FTY720 (1.5 mg/kg) significantly reduced the infarct size compared to that in the vehicle group (††*P* < 0.01 versus 1.5 mg/kg). **b** FTY720 showed a dose-dependent improvement in mortality ratio (vehicle, *n* = 36; 0.5 mg/kg, *n* = 19; 1.5 mg/kg, *n* = 12). **c**, **d** Neurological function was assessed by the modified Neurological Severity Score (**c**) and modified Bederson score (**d**) in the vehicle, low-dose FTY720, and high-dose FTY720 groups (vehicle, *n* = 24; 0.5 mg/kg, *n* = 12; 1.5 mg/kg, *n* = 12). Vehicle group, blue; 0.5 mg/kg group, red; 1.5 mg/kg, green. Significant differences between the vehicle and treatment groups. Values are the mean; *P < 0.05 versus 0.5 mg/kg, †*P* < 0.05 versus 1.5 mg/kg, **P < 0.01 versus 0.5 mg/kg, ††*P* < 0.01 versus 1.5 mg/kg

There was a dose-dependent reduction in infarct size between groups, and a significant difference was detected between the high-dose FTY720 group and vehicle group (*P* < 0.01); specifically, FTY720 reduced the infarct size by approximately 55% in the high-dose group compared to in the vehicle group (Fig. 1a). Mortality was also reduced in a dose-dependent manner; specifically, the vehicle group showed a rate of 20.0%, whereas the low-dose FTY720 group showed a rate of 8.0% and the high-dose group showed no mortality (Fig. 1b). A significant improvement in functional recovery was also observed in FTY720-treated animals, based on both the mNSS and modified Bederson Score, compared to in the vehicle group (*P* < 0.01). However, there was no difference in functional recovery between the low-dose and high-dose FTY720 groups (Fig. 1c, d).

### Activated Microglia/Macrophages and Apoptotic Cells Are Decreased in FTY720 Treatment Groups

**Fig. 2:**
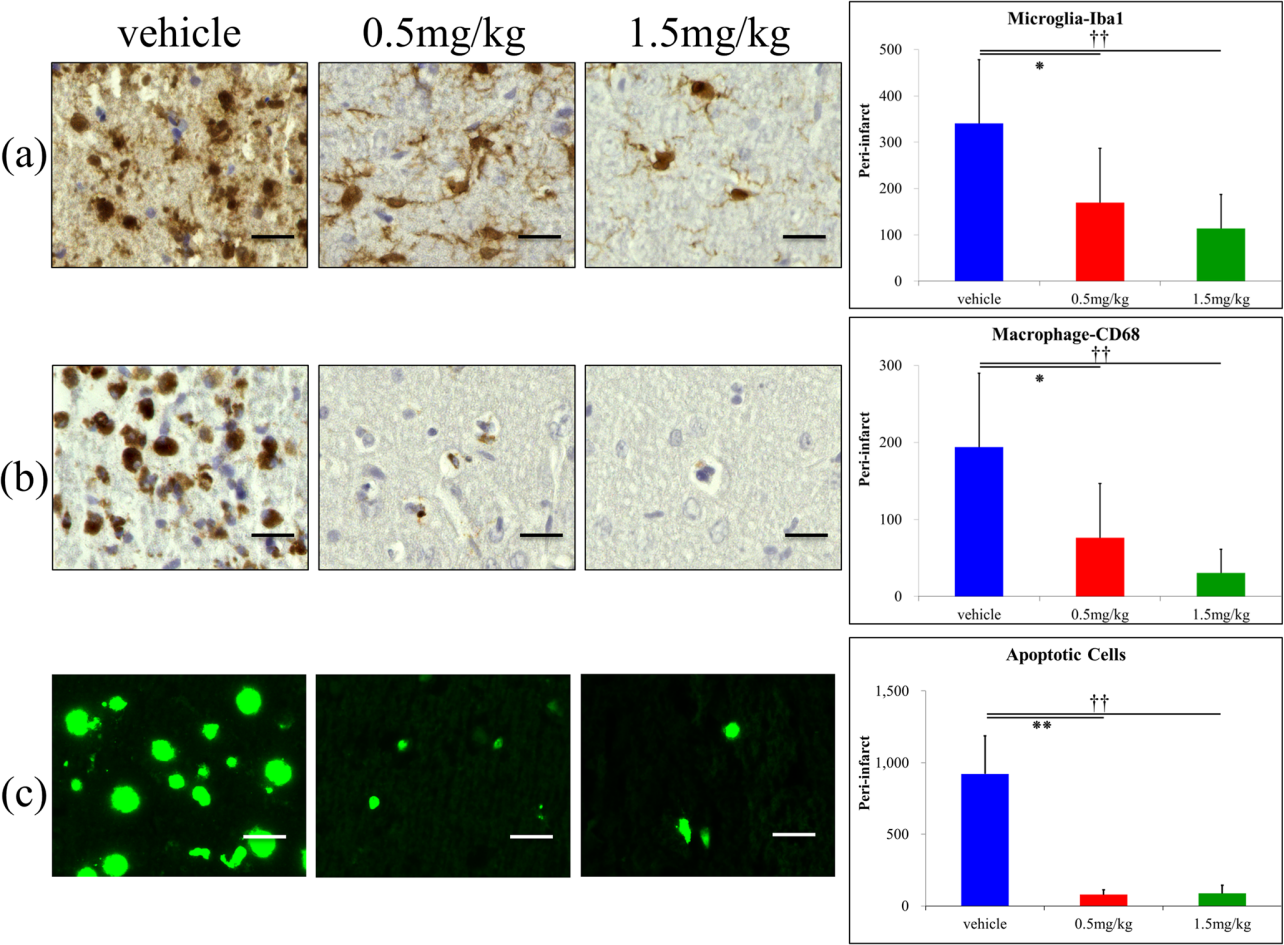
Immunohistochemistry staining (day 7) and TUNEL staining (day 3) of transient middle cerebral artery occlusion rat model showing the extent of inflammation and cell death including apoptosis in the peri-infarct areas. Iba-1-positive activated microglia (**a**) and macrophages (**b**) were dose-dependently decreased in the FTY720 groups (*n* = 6 per group); *P < 0.05 versus 0.5 mg/kg, ††*P* < 0.01 versus 1.5 mg/kg. FTY720 significantly reduced the number of cell death including apoptotic cells in the FTY720-treated groups (*n* = 6 per group); **P < 0.01 versus 0.5 mg/kg, ††*P* < 0.01 versus 1.5 mg/kg. Scale bar represents 25 μm. Higher magnification figure on the right upper corner (× 5)

Activated microglia identified by Iba1 staining were counted at the infarct border (Fig. 2a). Further, the number of activated microglia (as identified by dark Iba1 staining) was dose-dependently decreased in the FTY720 treatment groups. Specifically, the number of activated microglia in the low-dose group was decreased by 50.0% (*P* < 0.01) and that in the high-dose group was decreased by 64.4% (*P* < 0.01) compared to in the vehicle group. To investigate the effect of FTY720 on macrophage infiltration into damaged brains, CD68-positive cells were stained. Similar results were observed with Iba1-positive macrophages; specifically, FTY720 significantly decreased the number of CD68-positive macrophages by 60.6% (*P* < 0.01) in the low-dose group and by 84.2% (*P* < 0.01) in the high-dose group compared to in the vehicle group (Fig. 2b). Furthermore, TUNEL staining revealed that apoptotic cells were significantly decreased by approximately 90% in both the low-dose (91.4%, *P* < 0.01) and high-dose (90.5%, *P* < 0.01) FTY720 groups compared to the number in the vehicle group (Fig. 2c).

### PET Imaging Revealed Suppression of Inflammatory Reactions by FTY720 at the Subacute Phase

**Fig. 3:**
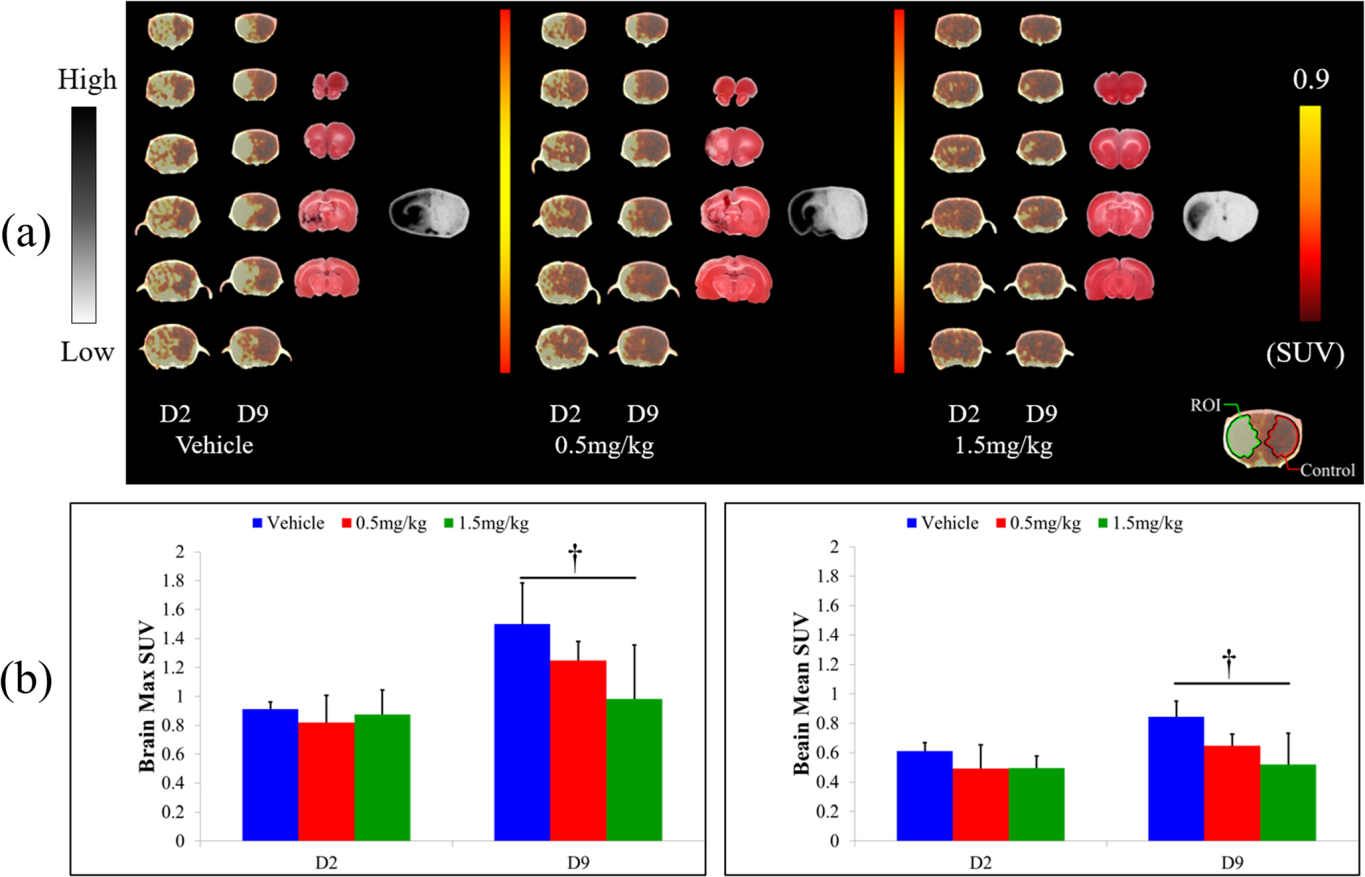
Serial PET imaging of inflammation in transient middle cerebral artery occlusion rat model. **a** Left figures show representative PET images (yellow: ischemic area; and brown: healthy area) (*n* = 4 per group), 3,5-triphenyltetrazolium chloride (TTC) staining (red represents healthy area and white represents ischemic area), and autoradiography (ARG; black represents ischemia and gray represents healthy area) of rat brains. Color bars indicate the scale of standardized uptake values (SUVs) in PET images (right) and qualitative scale in ARG (left). **b** Graphs show the max SUV (left) and the mean SUV (right); †*P* < 0.05 versus 1.5 mg/kg. Vehicle-treated animals showed a significant increase in inflammation on day 9 compared to that on day 2, whereas FTY720 suppressed this effect. Particularly, in the high-dose group (1.5 mg/kg), nearly no exacerbation of inflammation was observed

PET imaging was used to serially monitor the effect of FTY720 on inflammatory reactions (Fig. 3). [^18^F]DPA-714 PET showed no difference in inflammatory reactions among all groups 2 days after MCAO, as determined by the maximum (max) SUV and mean SUV (vehicle group: max SUV = 0.91 ± 0.05, mean SUV = 0.61 ± 0.06; low-dose FTY720 group: max SUV = 0.82 ± 0.19, mean SUV = 0.49 ± 0.16; high dose FTY720 group: max SUV = 0.88 ± 0.17, mean SUV = 0.50 ± 0.08). However, 9 days after MCAO, whereas the SUV was significantly increased in the vehicle group, a dose-dependent reduction was observed in the FTY720 treatment groups (vehicle group: max SUV = 1.50 ± 0.28, mean SUV = 0.84 ± 0.11; low-dose FTY720 group: max SUV = 1.25 ± 0.13, mean SUV = 0.65 ± 0.08; high-dose FTY720 group: max SUV = 0.98 ± 0.37, mean SUV = 0.52 ± 0.21). Notably, no exacerbation in inflammation was observed in the high-dose FTY720 group during the experiment.

### FTY720 Ameliorates BBB Disruption In Vivo and In Vitro Model of Ischemia

**Fig. 4:**
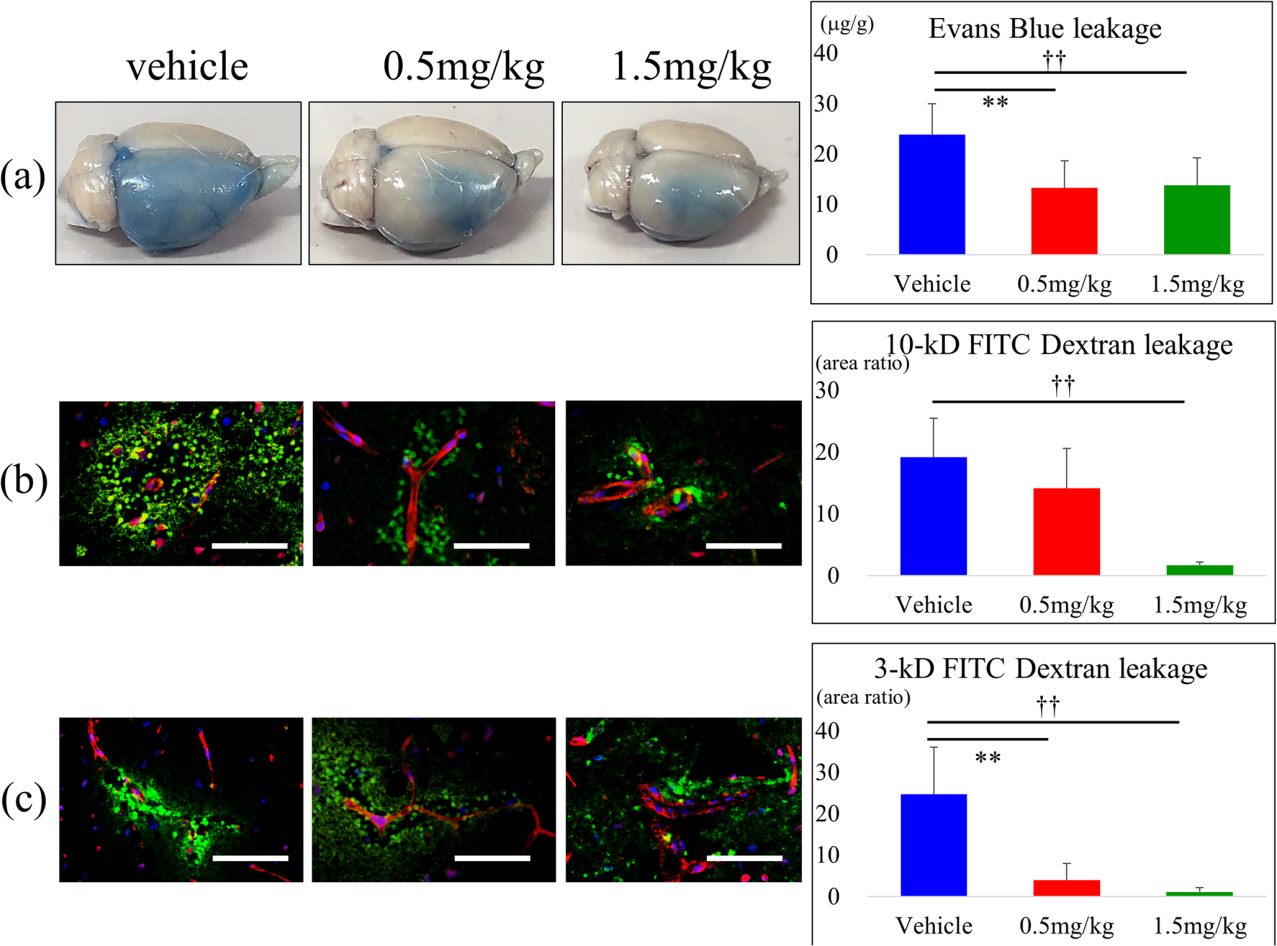
Effect of FTY720 on blood–brain barrier (BBB) permeability in the brain of ischemia–reperfusion (I/R rats). After middle cerebral artery occlusion (MCAO)/reperfusion, Evans blue, 10-kD fluorescent dextran (FITC dextran), or 3-kD FITC dextran was infused by i.v. injection at day 1. The brains were isolated, and the supernatant was measured at 620 nm for absorbance using a spectrophotometer. Representative pictures and the spectrophotometry optical density of Evans blue was compared between groups (**a**) (*n* = 6 per group). Immunohistochemistry staining of brain microvessels (red) and leaked FITC (green) were compared (**b**; 10-kD FITC dextran, **c**; 3-kD FITC dextran). Scale bar represents 50 μm. **P < 0.01 versus 0.5 mg/kg, ††*P* < 0.01 versus 1.5 mg/kg

**Fig. 5:**
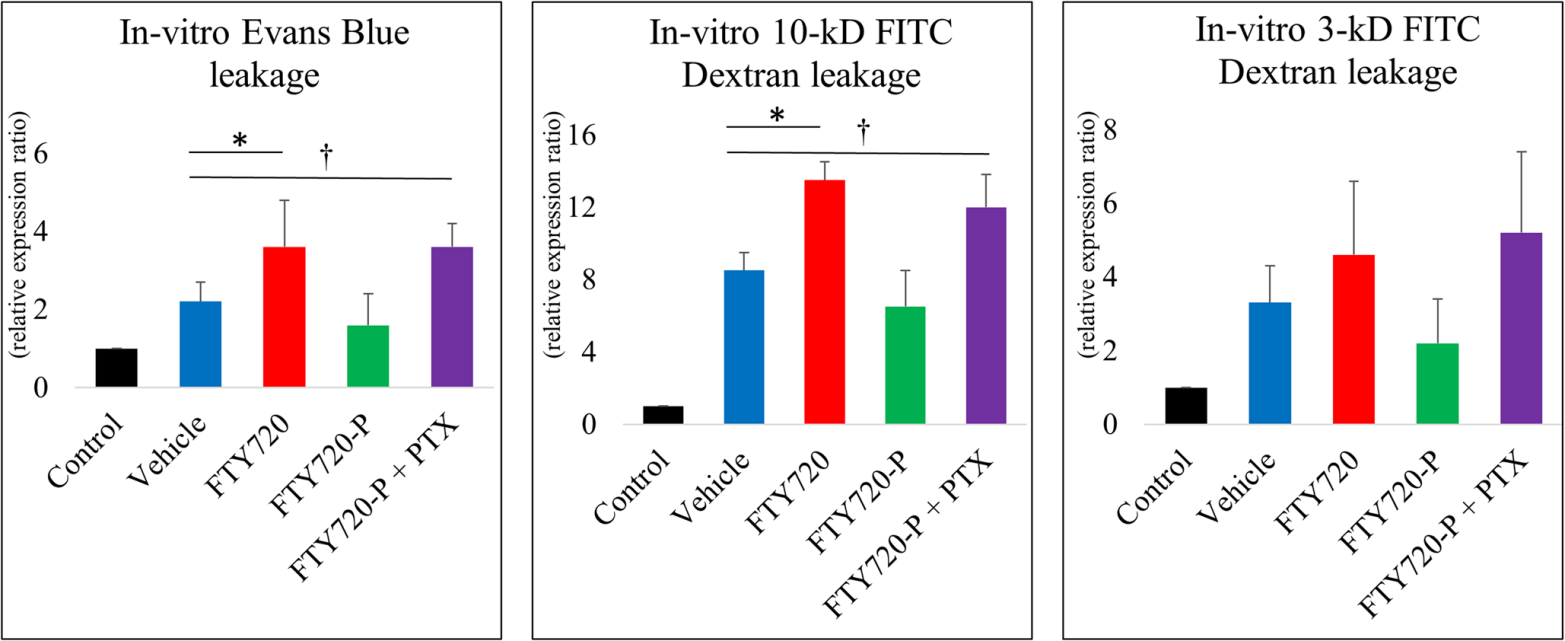
Effect of FTY720/FTY720-P on BBB permeability in in vitro ischemia model. Relative expression levels were examined in **a** Evans blue, **b** 10-kD FITC dextran, and **c** 3-kD FITC dextran. OGD condition (vehicle) disrupted BBB permeability compared to control. PTX appears to abolish the effect of FTY720-P. However, FTY720 or FTY720-P showed no protective role compared to OGD. FTY720 rather showed increased permeability in Evans blue and 10-kD FITC dextran compared to OGD. *P < 0.05 vehicle versus FTY720, †*P* < 0.05 vehicle versus FTY720-P + PTX

The effect of FTY720 against BBB disruption was assessed in both in vivo and in vitro model of ischemia. Evans blue extravasation was used to monitor in vivo rat brain BBB permeability for large molecule leakage. The content of dye in the ischemic hemisphere was significantly lower in both the high- and low-dose FTY720 groups compared to in the vehicle group (*P* < 0.01; Fig. 4a). Two different molecular weights of fluorescent dextran (3 and 10 kDa) were then used to assess BBB integrity for smaller molecules in the rat brain. The area ratio of fluorescent dextran extravascular leakage was dose-dependently decreased by both 3 and 10 kDa dextran (Fig. 4b, c). The effect of FTY720 and FTY720-P against BBB disruption was evaluated in an in vitro model. Leakage of Evans blue or FITC dextran was increased by the OGD condition (vehicle group). FTY720-P clearly ameliorated BBB permeability, although the difference was not significant. The effect of FTY720-P was completely abolished and even worsened by the existence of PTX (Fig. 5).

### FTY720/FTY720P Does Not Alter BBB mRNA Expression but Prevents Redistribution of BBB Components

**Fig. 6:**
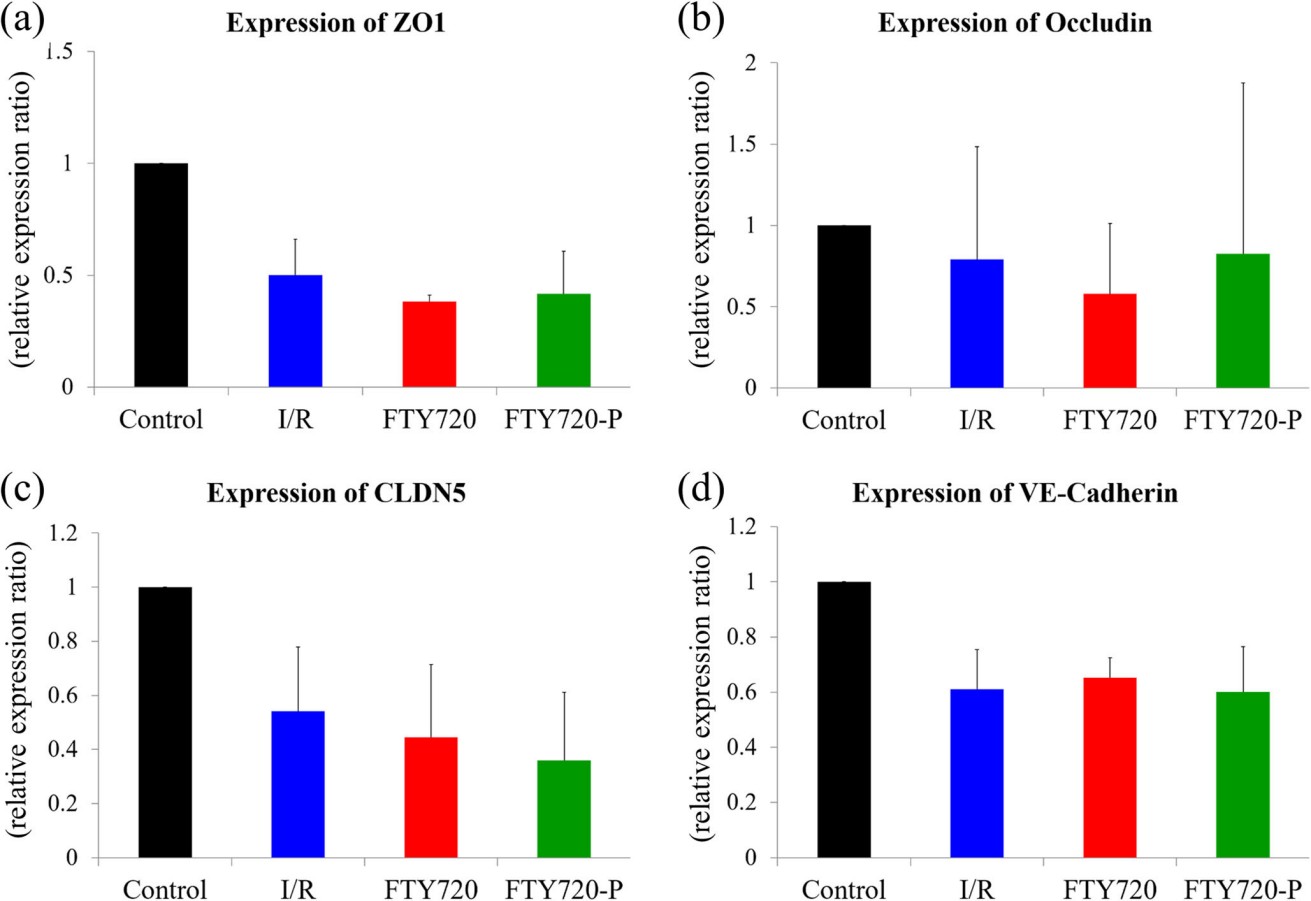
mRNA expression of tight and adherens junctional proteins in transient middle cerebral artery occlusion rat model. mRNA expression for *ZO-1* (**a**), *Occludin* (**b**), *Claudin-5* (*CLDN5*) (**c**), and *VE-cadherin* (**d**) were evaluated. Ischemia–reperfusion (I/R) injury resulted in decreased mRNA expression; however, FTY720 and FTY720P did not restore the expression of mRNA

**Fig. 7:**
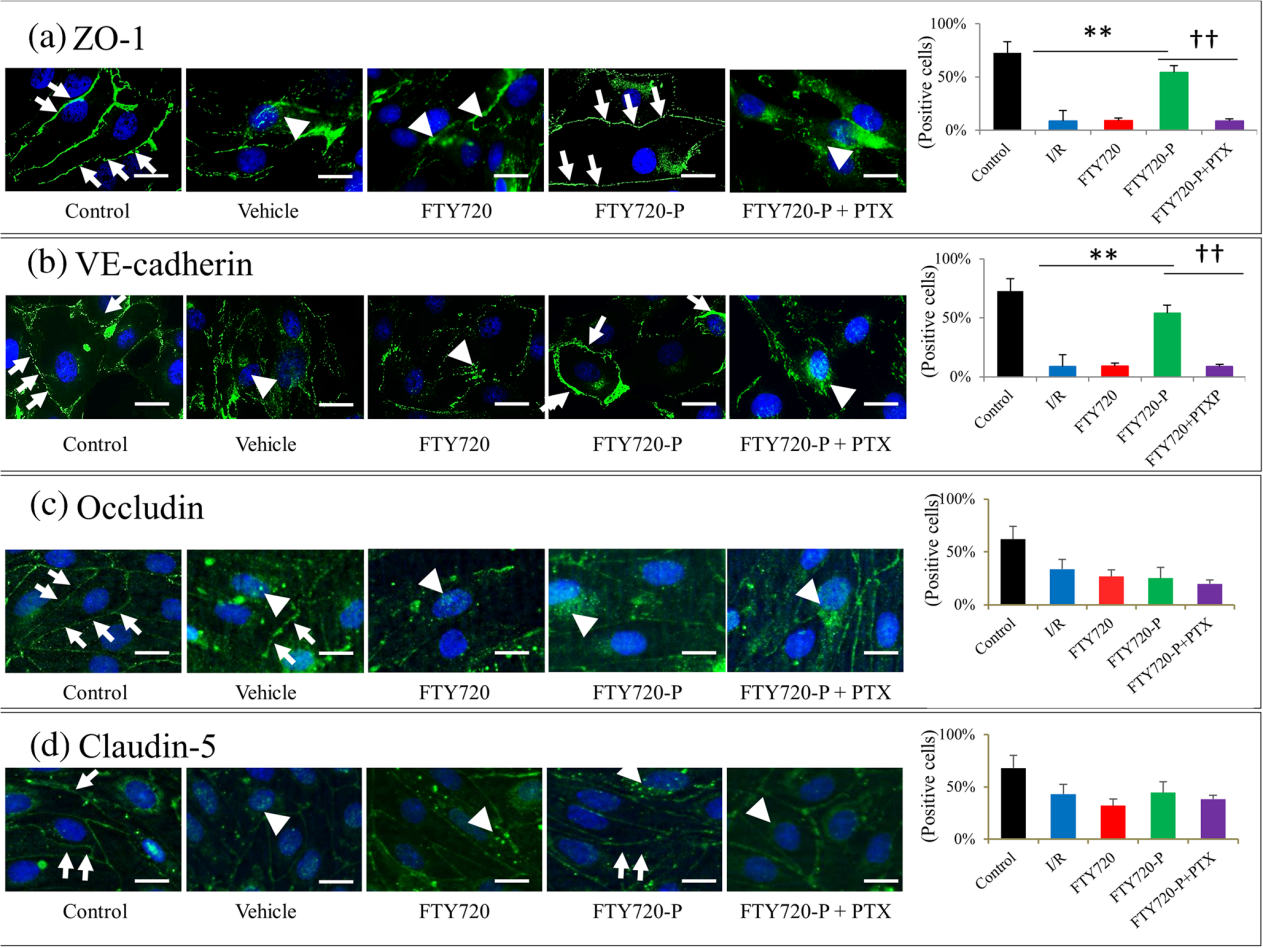
Translocation of tight and adherens junction proteins in transient middle cerebral artery occlusion rat model (**a** ZO-1, **b** VE-cadherin, **c** occludin, **d** Claudin-5). Whereas ZO-1 and VE-cadherin were distributed at the lamellipodia under physiological conditions (**a**, **b**; control, arrow), disruption and retraction of these proteins into the cytoplasm was observed after ischemia–reperfusion (I/R) injury (**a**, **b**; vehicle, arrowhead). While FYT720 groups (**a**, **b**; FTY720, arrowhead) showed the same results as the vehicle group, specifically that junctional proteins were not preserved at the lamellipodia, FTY720-P successfully maintained the junctional proteins similar to that observed in the control group (**a**, **b**; FTY720-P; arrow). This effect was completely abolished by adding the S1P_1_ receptor antagonist, pertussis toxin (PTX) (**a**, **b**; FTY720-P+PTX, arrowhead). These protective roles of FTY720P was not observed in the occludin (**c**) and claudin-5 (**d**). Scale bar = 10 μm. *P < 0.05 versus vehicle group, **P < 0.01 versus control group, ††*P* < 0.01 versus PTX+FTY720-P group

**Fig. 8:**
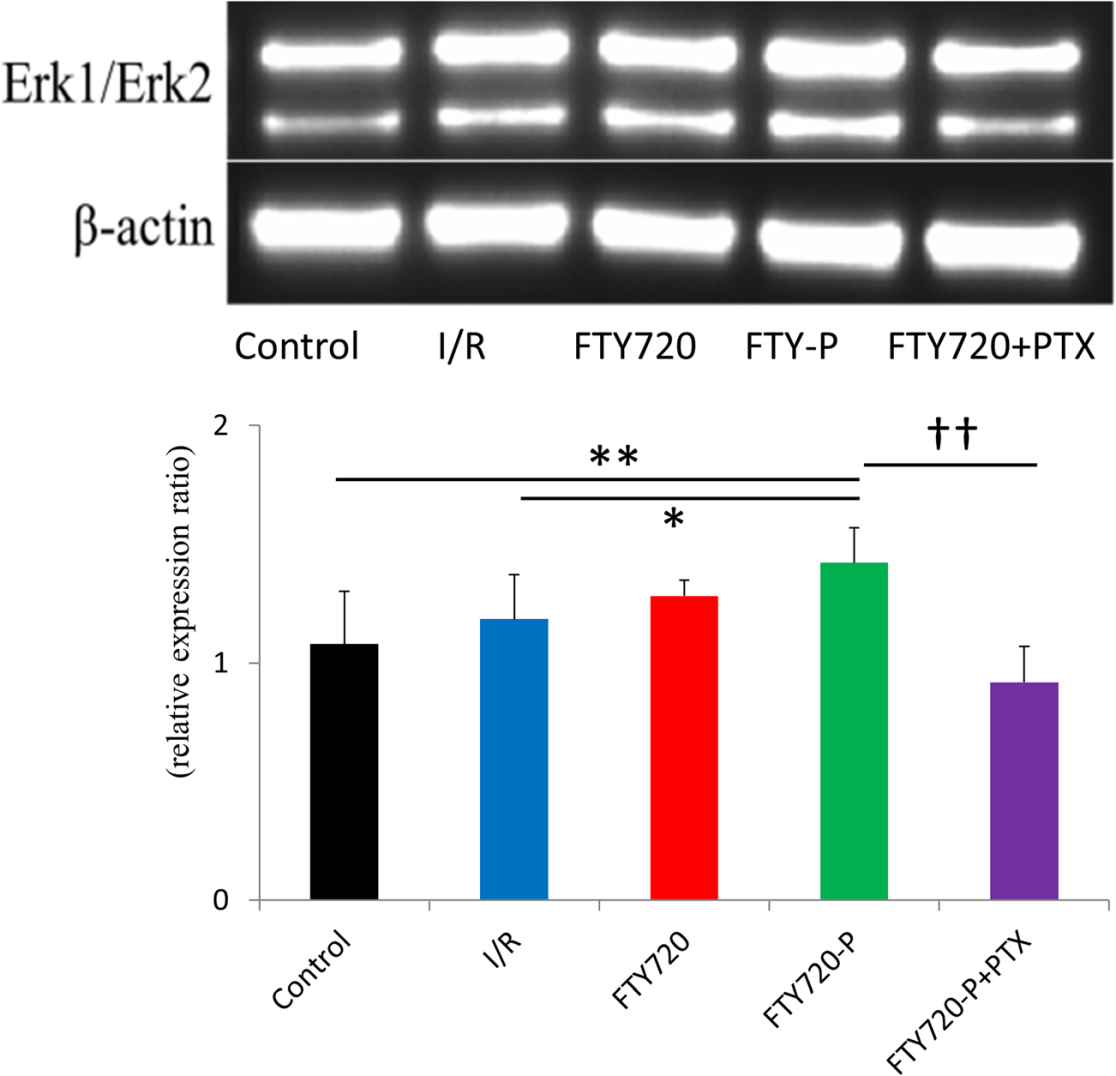
Western blot analysis was performed to elucidate the downstream signaling of FTY720-P; ERK1/2 signaling was significantly increased in the FTY720-P group and completely abolished by PTX treatment (*n* = 3 per group); *P < 0.05 versus vehicle group, **P < 0.01 versus control group, ††*P* < 0.01 versus PTX+FTY720-P group

Quantitative real-time PCR analysis of tight and adherens junction markers was performed to elucidate the effect of FTY720 on the BBB. BBMVECs were treated with I/R injury for further analysis. The results revealed decreased mRNA levels of tight junction (*ZO-1*, *occludin*, *claudin5*) and adherens junction (*VE-cadherin*) markers. However, FTY720 and FTY720-P could not rescue the expression of these components as compared to levels in the vehicle group (Fig. 6). Therefore, BBB proteins and their distributions were further investigated (Fig. 7). Immunofluorescence analysis revealed ZO-1 and VE-cadherin, occludin, and claudin-5 proteins at the junctional cell surface (lamellipodia) of BBMVECs/rat brain microvascular endothelial cells cultured under normal conditions (Fig. 6; control), whereas I/R injury led to the redistribution and retraction of these proteins into the endothelial cytoplasm, which was thought to result in BBB disruption (Fig. 6; vehicle). Whereas vehicle and FTY720 did not ameliorate the redistribution of BBB components, FTY720-P significantly preserved ZO-1 and VE-cadherin proteins at the cell lamellipodia (Fig. 6a, b). The effect of FTY720-P on the protection of BBB components at the lamellipodia was further confirmed by adding PTX, which antagonizes the major downstream pathway (Gi) of the S1P1 receptor; this completely abolished the effect of FTY720-P. In contrast, this preservation of junctional proteins was not observed for occludin and claudin-5 (Fig. 6c, d). We then determined whether the effect of FTY720-P occurred through S1P_1_ receptor binding. FTY720-P upregulated the expression of ERK1/2 signaling, which is downstream of the S1P_1_ receptor, as compared to that in the vehicle and FTY720 groups, whereas PTX again completely abolished downstream signaling from FTY720-P (Fig. 8).

## Discussion

In the present study, we identified two novel protective mechanisms of FTY720 against I/R injury. First, FTY720 protected BBB integrity by preventing the redistribution of lamellipodia-located tight and adherens junctions, ZO-1 and VE-cadherin into the cytoplasm, which was mediated by S1P_1_ receptor signaling. Second, FTY720 alleviated neuroinflammation not in the acute phase but rather in the subacute phase, which was confirmed by serial PET imaging. In addition, FTY720 treatment led to a decrease in infarct size, improved neurological function, and ameliorated microglial/macrophage activation and cell death.

During brain I/R injury, tight and adherens junction proteins are disrupted, which leads to a compromised BBB, allowing T and B cells to cross and subsequently causing inflammatory reactions [5, 21]. FTY720 has shown to stimulate the assembly of BBB junctional proteins such as VE-cadherin and β-catenin under physiological conditions through Gi-coupled S1P receptors [32]. A previous report revealed that S1P administration induces the translocation of junction or adherens proteins into the lamellipodia and junctional regions, thereby enhancing barrier integrity [33]. These observations are in line with our findings showing that junctional proteins of ZO-1 and VE-cadherin are preserved from I/R injury-induced translocation to the cytoplasm, resulting in BBB preservation. We found that FTY720 does not upregulate the mRNA expression of junctional proteins, suggesting that the de novo synthesis of junctional proteins was not the reason for the observed BBB preservation. Furthermore, the redistribution of these tight and adherens junctional proteins was abolished by PTX, which is the Gi receptor antagonist and main mediator of downstream signaling from the S1P_1_ receptor. This strongly suggests that this BBB protective mechanism is executed by S1P_1_ receptor signaling. However, unexpectedly, the protein distribution of occludin and claudin-5 were not preserved by FTY720-P. These differences may have occurred because junctional proteins have different functional properties [34], but further studies are necessary to confirm this difference. Cannon et al. have previously reported that FTY720 acts on the brain endothelial cell S1P receptor 1 to reduce basal P-glycoprotein (P-gp), which functions as a ATP-driven drug efflux pump [35]. Our results and those of Cannon et al. suggest that TY720 prevents tight junction disruption at the expense of reducing downregulation of P-gp transport. Our data suggest a new mechanism by which FTY720 protects against ischemic insults, in which FTY720 preserves the BBB tight and adherens junction on the cell lamellipodia and prevents them from redistributing to the cytoplasm. In contrast, there have been conflicting reports, suggesting that co-administration of FTY720 and recombinant tissue plasminogen activator (rt-PA) can exacerbate BBB disruption [36], or that TEER of endothelial cells exposed to IFNγ and TNFα were not changed by FTY720-P administration [37]. Reports from Hla et al. also showed that FTY720 facilitates selective BBB permeability to small molecules under physiological conditions. These conflicting data indicate the complicated mechanisms associated with BBB function and role of FTY720 [38]. S1P is biosynthesized from ceramide and sphingosine, and the expression levels of these sphingolipids are strongly related [39, 40]. Because ceramide, sphingosine, and S1P show unique effect on cells, further investigation of sphingolipids against BBB protection is necessary to determine the BBB regulatory and functional mechanisms.

Another major finding of this study was that FTY720 can ameliorate inflammation not during the acute phase but rather during the subacute stage, which was demonstrated by serial imaging of inflammation using the same animal via PET. TSPO is known to be mainly located on the outer mitochondrial membrane [41] and has recently received attention because of its roles in multiple processes including immunomodulation, steroid synthesis, and apoptosis [42]. It is maintained at low levels in the central nervous system under normal physiological conditions but is highly expressed in activated microglia in response to brain injury and inflammation and may be a good marker of neuroinflammation [39, 43]. We found that vehicle-treated animals showed a significant increase in inflammation on day 9 compared to on day 2, whereas FTY720 suppressed this effect. Particularly, in the high-dose group (1.5 mg/kg), nearly no exacerbation of inflammation was observed. This result may be used to optimize the timing to evaluate the inflammatory response with FTY720 in the future and may be a biomarker of successful treatment. The reason for the observed attenuation in inflammation at the later stage remains unclear, as inflammation is mediated by many critical factors including lymphocytes, leukocytes, microglia/macrophages, and the BBB. Lymphopenia has been reported as a primary mechanism for FTY720 both in vitro and in vivo and is considered one of the most important therapeutic targets for this drug [15]. Administration of FTY720 in the early stage of ischemic stroke is clearly beneficial, as it induces lymphopenia, but this agent is considered to have a shorter half-life; thus, we suggest that this is not the only reason for this effect. We showed that FTY720 significantly reduces the number of activated microglia/macrophages and apoptotic cells. Interestingly, macrophages were reported to express the S1P_2_ receptor, whereas lymphocytes mainly harbor the S1P_1_ receptor [40]. FTY720 acts on four of five S1P receptors (S1P1, S1P3, S1P4, S1P5), demonstrating that it does not directly act on microglia. FTY720 was also reported to show no direct anti-apoptotic effect on neural cells after ischemic insults [17]. Our result indicates that FTY720 significantly reduces microglia/macrophage activation and cell death including apoptosis, not through the direct action of FTY720 on damaged cells, but rather through secondary damage in association with other factors including leukopenia and BBB preservation. This may also be confirmed by the fact that FTY720 could not improve outcomes following permanent MCAO but required reperfusion [44].

There were several limitations to this study. First, we were able to determine whether FTY720-P prevents the redistribution of tight and adherens junctional proteins, ZO-1 and VE-cadherin, to the lamellipodia, and FTY720 ameliorates BBB leakage in vivo. However, we were not able to directly demonstrate the relationship between the redistribution of BBB proteins and BBB leakage. Electron microscopy of the junctional regions or electrical cell substrate impedance assays, as well as measuring protease activities including MMP-9 may help to resolve this issue. Second, we could not directly demonstrate that preserving the BBB was associated with other results such as smaller infarct size, lower inflammation, and cell death. As lymphopenia is considered as one of the most prominent mechanisms underlying neurological preservation through FTY720, gene knockout mouse models or models in which leukocytes are abolished may be required to more precisely determine the effect of FTY720 on the BBB. Third, the drug was i.p.-injected from a technical perspective; however, when considering clinical use, i.v. injection and oral drug administration should also be applied. Fourth, FTY720 has increasingly been recognized to exert epigenetic effects independent of S1P receptor signaling [45–49]. We could not track the epigenetic effect of FTY720 in this study, particularly as it relates to ameliorating BBB functional deficiencies. Chromatin immunoprecipitation may be necessary to reveal this association.

In conclusion, the present study suggests that FTY720 can ameliorate I/R injury, protecting the BBB by preventing the redistribution of junctional proteins into the cytoplasm and suppressing neuroinflammation at the subacute stage. Thus, FTY720 may be a promising candidate for alleviating I/R injury after revascularization.

## Electronic Supplementary Material

ESM 1 (XLSX 9 kb)
